# The Suppressive Effect of Resveratrol on HIF-1α and VEGF Expression after Warm Ischemia and Reperfusion in Rat Liver

**DOI:** 10.1371/journal.pone.0109589

**Published:** 2014-10-08

**Authors:** Mei Zhang, Wujun Li, Liang Yu, Shengli Wu

**Affiliations:** 1 Department of Hepatobiliary Surgery, the First Affiliated Hospital of Xi'an Jiaotong University, Xi'an, P.R. China; 2 Department of General Surgery, the First Affiliated Hospital of Xi'an Medical University, Xi'an, P.R. China; Indian Institute of Toxicology Reserach, India

## Abstract

**Background:**

Hypoxia-inducible factor-1α (HIF-1α) is overexpressed in many human tumors and their metastases, and is closely associated with a more aggressive tumor phenotype. The aim of the present study was to investigate the effect of resveratrol (RES) on the expression of ischemic-induced HIF-1α and vascular endothelial growth factor (VEGF) in rat liver.

**Methods:**

Twenty-four rats were randomized into Sham, ischemia/reperfusion (I/R), and RES preconditioning groups. I/R was induced by portal pedicle clamping for 60 minutes followed by reperfusion for 60 minutes. The rats in RES group underwent the same surgical procedure as I/R group, and received 20 mg/kg resveratrol intravenously 30 min prior to ischemia. Blood and liver tissue samples were collected and subjected to biochemical assays, RT-PCR, and Western blot assays.

**Results:**

I/R resulted in a significant (*P*<0.05) increase in liver HIF-1α and VEGF at both mRNA and protein levels 60 minutes after reperfusion. The mRNA and protein expressions of HIF-1α and VEGF decreased significantly in RES group when compared to I/R group (*P*<0.05).

**Conclusion:**

The inhibiting effect of RES on the expressions of HIF-1α and VEGF induced by I/R in rat liver suggested that HIF-1α/VEGF could be a promising drug target for RES in the development of an effective anticancer therapy for the prevention of hepatic tumor growth and metastasis.

## Introduction

Hepatocellular carcinoma (HCC) is one of the most common malignancies in the world [1]. Surgical resection and liver transplantation are conventional treatment modalities that can offer long-term survival for patients with HCC. However, the high incidence of tumor recurrence and metastasis after liver surgery remains a major problem [2]. Hepatic ischemia/reperfusion (I/R) injury is a phenomenon inevitable during liver surgery and promotes liver tumor growth and metastases through activation of cell adhesion, invasion, and angiogenesis pathways [3]. Hypoxia-inducible factor-1 alpha (HIF-1α) is one of the key regulators of hypoxia/ischemia [4]. Accumulating evidence indicated that the outgrowth of hepatic micrometastases is stimulated by I/R injuries during surgery and may at least in part, be stimulated by an increased HIF-1α stabilization [5], [6]. HIF-1α stimulates transcription of multiple genes, including angiogenic vascular endothelial growth factor (VEGF) [7], an important growth factor involved in tumor angiogenesis [8], and HIF-1α/VEGF pathway have been implicated in the development of multiple tumors [9]–[11].

Resveratrol (trans-3,4′,5-trihydroxystilbene, RES) is a natural polyphenolic phytoalexin found in various plant species [12]. Numerous studies have demonstrated its diverse pharmacological activities, including antitumor and chemopreventive properties [13]. Studies in animal models have demonstrated that RES exerts potent anticarcinogenic effects via affecting diverse cellular events associated with tumor initiation, promotion, and progression [14]. Recently, RES has been found to inhibit angiogenesis and its antiangiogenic effects had been investigated in the setting of in vitro hypoxia, but the underlying mechanism of its antiangiogenic activity remains unclear [15].

In this study, we aimed to investigate whether RES inhibited I/R induced HIF-1α accumulation and VEGF expression in a rat model. The findings will provide further evidence that RES can be a potential chemopreventive and anticancer agent for reducing liver tumor recurrence and metastasis after liver surgery for HCC patients.

## Materials and Methods

### Animals

Male Sprague-Dawley (SD) rats 9–10 weeks old weighing 190–210 g were purchased from the Animal Center of Xi'an Jiaotong University (Xi'an, China). All rats were allowed free access to water and standard laboratory chow. Before operation the rats were fasted for 12 h and only allowed free access to water. Care was provided in accordance with the “Guide for the care and use of laboratory animals” (NIH publication No. 85–23, revised in 1996). The study was approved by the Xi'an Jiaotong University Institutional Animal Care and Use Committee.

### Reagents

Resveratrol and dimethyl sulfoxide (DMSO) were purchased from Sigma Chemical Co., USA. RPMI-1640 was from Gibco-BRL, USA. The RES was dissolved and sterilized in DMSO and then diluted in RPMI-1640 to 4 mg/mL.

### Experimental design

Rats were anesthetized with an intraperitoneal injection of pentobarbital sodium (50 mg/kg; Nembutal, Abbott Laboratories, North Chicago, IL). Twenty-four rats were randomly divided into three experimental groups (eight rats in each group) as follows: Sham operation group, a 6-cm midline abdominal incision was made to expose the liver and laparotomy was carried out for 60 min with no hepatic ischemia; I/R group: I/R was induced by portal pedicle clamping with an atraumatic microvascular clip for 60 minutes followed by removal of the clip for 60 minutes, and rats received an equivalent volume of placebo solution (RPMI-1640); and RES preconditioning group: rats in this group underwent the same surgical procedure as I/R group, and received one-shot injection of RES (4 mg/mL) at a dose of 20 mg/kg body weight through vena dorsalis penis 30 min prior to ischemia. All rats were euthanized with an overdose of pentobarbital (100 mg/kg IV) followed by exsanguinations at 60 min after clip removal, while Sham operation group animals were killed at the same time points after surgery. The liver was removed, and the inferior vena cava was cannulated so that blood samples could be taken from its suprahepatic segment. Blood samples were centrifuged at 4000 r/min at 4°C for 3 min and serum was taken and immediately processed. Liver tissues were snap-frozen in liquid nitrogen and stored at −80°C for further analysis.

### Measurement of Serum Liver Enzymes

In all three groups serum alanine aminotransaminase (ALT), alkaline phosphates (ALP), and total bilirubin (TBIL) concentrations were measured using Hitachi AU5400 automatic biochemical analyzer (Hitachi Corp., Japan) and Roche Diagnostics kit (Roche, USA) at 60 min after reperfusion.

### Real-time RT-PCR

After homogenization of liver tissue by the use of a MM301 Mixer Mill (Retsch, Haan, Germany), total cellular RNA was extracted from the liver tissue by using TriPure Reagent Isolation Reagent (Roche). RNA concentration was determined using UV spectrophotometer. Five hundred nanograms of RNA were reverse-transcribed and amplified to cDNA using real time RT-PCR with iScript One-Step RT-PCR Kit with SYBR Green (BioRad, USA). β-actin gene was used as an internal control. Primer sequences used in this study were designed using NCBI Primer-Blast (http://www.ncbi.nlm.nih.gov/tools/primer-blast) as follows: for the HIF-1α, sense 5′-ACTGCACAGGCCACATTCAT-3′ and antisense 5′-CGAGGCTGTGTCGACTGAGA-3′; for the VEGF, sense 5′-AGGCGAGGCAGCTTGAGTTA-3′ and antisense 5′-CTGTCGACGGTGACGATGGT-3′; for the β-actin, sense 5′-CCTAGGCACCAGGGTGTGAT-3′ and antisense 5′-TTGGTGACAATGCCGTGTTC-3′. The initial denaturation phase was 3 min at 95 °C followed by 39 cycles of denaturation at 95 °C for 10 s and annealing at 55 °C for 30 s. Relative quantification of PCR products was performed after normalization to β-actin.

### Western blot analysis

After homogenization of liver tissue by the use of a MM301 Mixer Mill (Retsch, Haan, Germany), total cellular protein was extracted from the liver tissue by using tissue protein extraction buffer (Pierce, Rockford, IL, USA) containing protease inhibitors (Protease Inhibitor Cocktail 100X, Pierce). Protein concentrations were determined and the samples were subjected to sodium dodecyl sulfate/polyacrylamide gel electrophoresis and transferred to a nitrocellulose membrane (ECL, Amersham, Buckinghamshire, UK). The membranes were then blocked for 60 min and subsequently incubated with primary antibodies (1∶3000) overnight at 4°C prior to incubation with anti-mouse IgG conjugated to horseradish peroxidase (1∶6000) for 120 min at room temperature. Finally, the signals were detected using an enhanced chemiluminescence detection kit (Amersham, Piscataway, NJ, USA). The chemiluminescent signal was captured by a UVP BioSpectrum500 imaging system (UVP, Upland, CA, USA). Protein expression was quantified by densitometry and normalized to β-actin expression. Anti-HIF-1α, anti-VEGF, and anti-β-actin antibodies were obtained from Santa Cruz Biotechnology, Inc. (Santa Cruz, CA, United States).

### Statistical analysis

All data are presented as the means ± standard deviation of the mean. Statistical analysis was performed using SPSS 16.0 software. Differences among groups were tested by one-way analysis of variance (ANOVA) with post-hoc Student-Newman-Keuls method. A *P*-value <0.05 was considered to indicate a statistically significant result.

## Results

### Liver function

Serum ALT, ALP and TBIL concentrations in different groups are shown in Table. 1. At 60 min post reperfusion, serum ALT, ALP and TBIL levels were significantly higher in I/R group than in the sham operation group (all *P*<0.05). Pretreatment with RES (20 mg/kg) showed a significant decrease in levels of serum ALT, ALP and TBIL than in I/R group (all *p*<0.05).

**Table 1:** Serum biochemical parameters in different groups (mean ±SD).

Groups	n	ALT (U/L)	ALP(U/L)	TBIL (umol/L)
Sham	8	43.85±15.64	85.39±21.65	6.57±1.12
I/R	8	1465.50±316.37*	415.71±68.43*	35.41±5.87*
RES	8	837.65±205.53**	297.42±23.44**	21.47±3.69**

*p<0.05 *vs* Sham operation group;

**p<0.05 *vs* I/R group.

### HIF-1α expression

**Figure 1:**
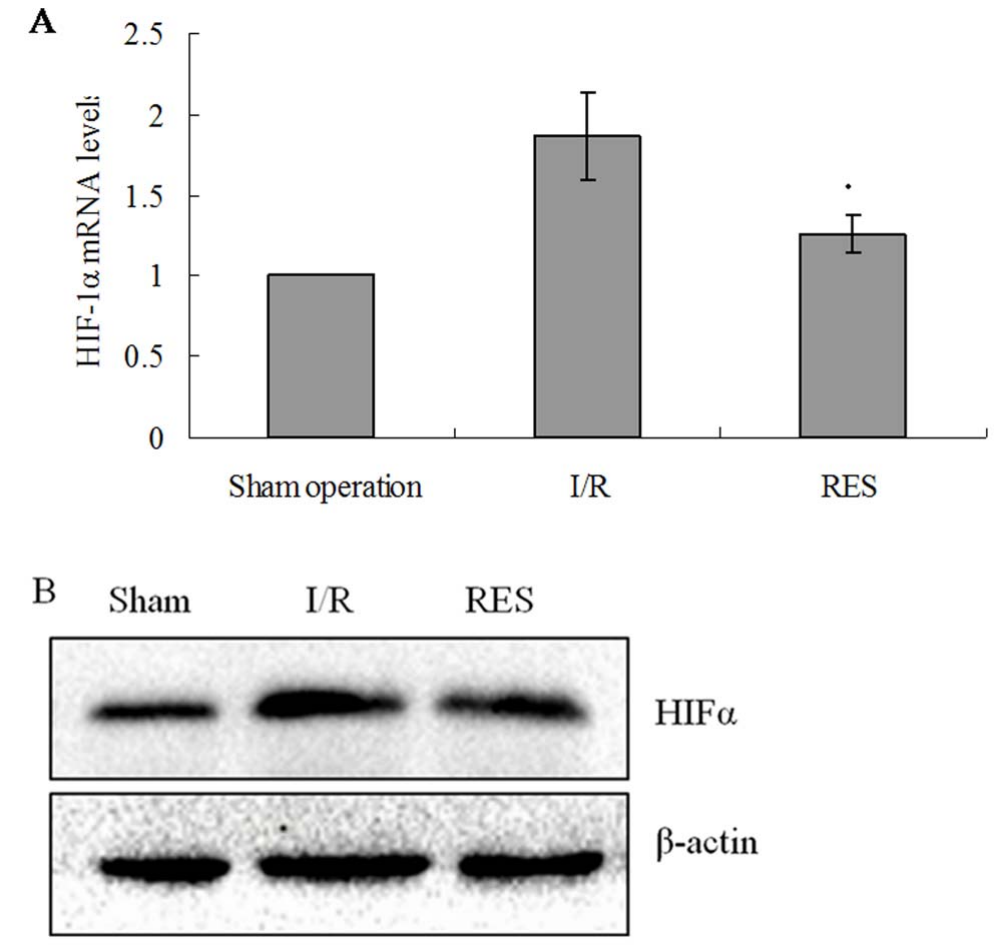
Expression of HIFα mRNA in rat livers. (A) HIFα mRNA levels were determined by real-time RT-PCR. Relative fold induction for HIFα mRNA (means ±SD) in I/R and RES group rat livers is presented relative to the expression in Sham operation group rat livers (*P<0.05 compared with I/R group). (B) Western blot analysis for HIFα protein expression in the indicated groups. β-actin was used as a loading control.

The expression of HIF-1α in livers of experimental rats was examined by real-time RT-PCR and western blotting methods. Compared to sham operation group, both mRNA and protein expressions of HIF-1α were significantly increased in the livers of rats in I/R group (all *P*<0.05). Compared to I/R group, a significant reduction in HIF-1α mRNA and protein expression levels in RES preconditioning group was observed (all *P*<0.05; Fig. 1A and B).

### VEGF expression

**Figure 2:**
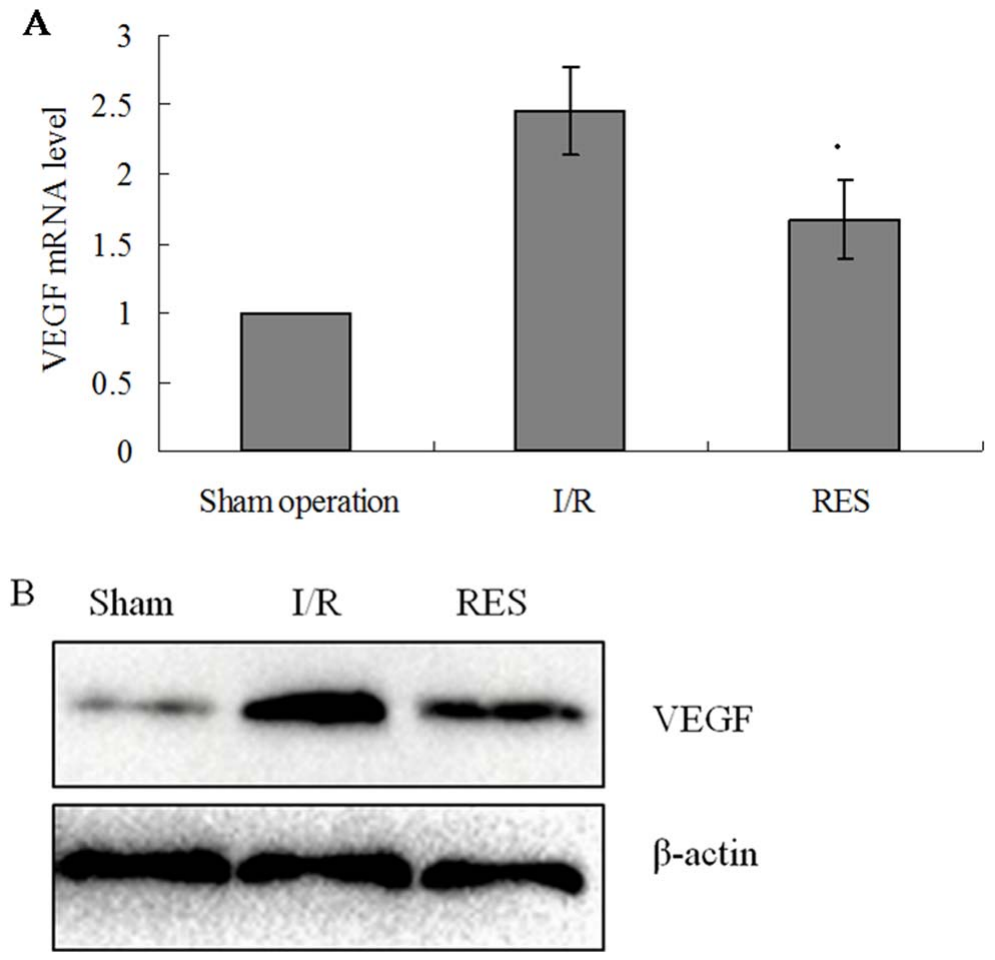
Expression of VEGF mRNA in rat livers. (A) VEGF mRNA levels were determined by real-time RT-PCR. Relative fold induction for VEGF mRNA (means ± SD) in I/R and RES group rat livers is presented relative to the expression in Sham operation group rat livers (*P<0.05 compared with I/R group). (B) Western blot analysis for VEGF protein expression in the indicated groups. β-actin was used as a loading control.

The expression of VEGF in livers of experimental rats was examined by real-time RT-PCR and western blotting methods. Compared to sham operation group, both mRNA and protein expressions of VEGF were significantly increased in the livers of rats in I/R group (all *P*<0.05). Compared to I/R group, a significant reduction in VEGF mRNA and protein expression levels in RES preconditioning group was observed (all *P*<0.05; Fig. 2A and B).

## Discussion

Hepatic I/R injury may cause metabolic and structural hepatic damage [16] and has been proposed as a key clinical problem associated with liver transplantation and major liver surgery [17]. It involves a complex series of events, such as mitochondrial deenergization, adenosine-5′-triphosphate depletion, alterations of electrolyte homeostasis, as well as Kupffer cell activation, oxidative stress changes and upregulation of proinflammatory cytokine signaling [18]. At the same time, cellular response to low tissue oxygen concentrations is mediated by HIF-1 to protect liver from I/R injury.

HIF-1 is composed of HIF-1α and HIF-1β subunits [19]. HIF-1α was firstly described by Semenza in 1992 and the expression of which is tightly regulated by low oxygen tension [20], whereas HIF-1β is constitutively expressed. Under normoxic conditions, HIF-1α protein is induced and continuously degraded by the ubiquitin-proteasome pathway in the cytoplasmic cellular compartment. However, under hypoxic conditions the blockade of degradation lead to the remarkable accumulation and translocation of HIF-1α protein to the nucleus, where it heterodimerizes with HIF-1β. This HIF-1 complex then initiates transcriptional activation via binding with hypoxia responsive elements in the promoter regions of target genes [21], including VEGF, erythropoietin, glycolytic enzymes, transferrin and a variety of other proteins that are important for adaptation and survival under hypoxic stress [22].

VEGF, an immediate downstream target gene of HIF-1α, plays a pivotal role in tumor angiogenesis [23], especially under conditions of intratumoral hypoxia. It promotes the proliferation of vessel endothelial cells, inhibits the apoptosis of vessel endothelial cells, and stimulates the formation of blood vessels [20]. Furthermore, it stimulates the production of hepatocyte growth factor (HGF), which is regarded as an initiator of liver regeneration [24]. Therefore, a stimulation of HIF-1α via liver ischemia, could be a double-edged sword; i.e., it protects the liver against I/R injuries, but a side effect could be the promotion of recurrence and metastasis of HCC through angiogenesis.

RES has been reported to have several biologic effects such as a potent antioxidative effect via prevention of lipid peroxidation, anti-platelet activity, an estrogenic activity, and anti-inflammatory activity attributed to cyclooxgenase inhibition [25]. Previous study had reported the hepatoprotective effects of RES in hepatic I/R and the protective effects of RES may be associated with its antioxidant activity and free radical scavenging activity which are released during the reperfusion period [26]. In recent years, RES has been found to inhibit tumor angiogenesis [27], but the mechanism of its antiangiogenic activity remains to be elucidated. Yu et al. reported that RES inhibits VEGF expression of HepG2 cells through a NF-kappa B-mediated mechanism [28]. Cao et al. reported that RES may inhibit human ovarian cancer progression and angiogenesis by inhibiting HIF-1α and VEGF expression through multiple mechanisms, including the inhibition of AKT and mitogen-activated protein kinase activation, the inhibition of several protein translational regulators, and inducing HIF-1α protein degradation through the proteasome pathway [29]. Zhang et al. showed that RES directly inhibits hypoxia-mediated HIF-1α protein accumulation by inhibiting its degradation via the proteasomal pathway in both SCC-9 and HepG2 cells [30]. In the present study, as expected, serum ALT, ALP and TBIL levels were significantly higher in I/R group than in the sham operation group at 60 min post reperfusion, while pretreatment with RES (20 mg/kg) showed a significant decrease in levels of serum ALT, ALP and TBIL than in I/R group. Moreover, we further showed that the mRNA and protein expressions of HIF-1α and VEGF were increased significantly in rats subjected to 60 minutes of warm liver ischemia and 60 minutes of reperfusion compared to the control group, while the mRNA and protein expressions of HIF-1α and VEGF decreased significantly in RES group when compared to I/R group. These findings affirmed the results of previous studies showing the hepatoprotective effects of RES in hepatic I/R. More importantly, we provided the first evidence supporting the antiangiogenic effects of RES in the setting of in vivo hypoxia, which are consistent with previous findings, suggesting that RES inhibits angiogenesis at least partly through regulating the expressions of HIF-1α and VEGF. However, additional studies are needed to identify the detailed mechanisms by which RES regulated the expressions of HIF-1α and VEGF.

Taken together, our present study has provided evidence that RES, exerts its antiangiogenic effects through inhibiting HIF-1α and its downstream target gene, VEGF, in a rat model of hepatic I/R injury. HIF-1α/VEGF axis, as a key regulator of tumor growth and metastasis, could be a promising drug target for RES in the development of an effective anticancer therapy for the prevention of hepatic tumor growth and metastasis.

## References

[1] Bosch FX, Ribes J, Díaz M, Cléries R (2004) Primary liver cancer: worldwide incidence and trends. Gastroenterology 127: S5–S16. 15508102 10.1053/j.gastro.2004.09.011

[2] Zhang Y, Shi ZL, Yang X, Yin ZF (2014) Targeting of circulating hepatocellular carcinoma cells to prevent postoperative recurrence and metastasis. World J Gastroenterol 20: 142–147. 24415867 10.3748/wjg.v20.i1.142 PMC3886003

[3] Li CX, Shao Y, Ng KT, Liu XB, Ling CC, et al (2012) FTY720 suppresses liver tumor metastasis by reducing the population of circulating endothelial progenitor cells. PLoS One 7: e32380. 22384233 10.1371/journal.pone.0032380 PMC3288101

[4] Cursio R, Miele C, Filippa N, Van Obberghen E, Gugenheim J (2008) Liver HIF-1 alpha induction precedes apoptosis following normothermic ischemia-reperfusion in rats. Transplant Proc 40: 2042–2045. 18675125 10.1016/j.transproceed.2008.05.037

[5] van der Bilt JD, Kranenburg O, Nijkamp MW, Smakman N, Veenendaal LM, et al (2005) Ischemia/reperfusion accelerates the outgrowth of hepatic micrometastases in a highly standardized murine model. Hepatology 42: 165–175. 15962318 10.1002/hep.20739

[6] Knudsen AR, Kannerup AS, Grønbæk H, Andersen KJ, Funch-Jensen P, et al (2011) Effects of ischemic pre- and postconditioning on HIF-1α, VEGF and TGF-β expression after warm ischemia and reperfusion in the rat liver. Comp Hepatol 10: 3. 21771288 10.1186/1476-5926-10-3 PMC3155899

[7] Boros P, Tarcsafalvi A, Wang L, Megyesi J, Liu J, et al (2001) Intrahepatic expression and release of vascular endothelial growth factor following orthotopic liver transplantation in the rat. Transplantation 72: 805–811. 11571441 10.1097/00007890-200109150-00011

[8] Tamagawa K, Horiuchi T, Uchinami M, Doi K, Yoshida M, et al (2008) Hepatic ischemia-reperfusion increases vascular endothelial growth factor and cancer growth in rats. J Surg Res 148: 158–163. 18468635 10.1016/j.jss.2007.12.787

[9] Chai ZT, Kong J, Zhu XD, Zhang YY, Lu L, et al (2013) MicroRNA-26a inhibits angiogenesis by down-regulating VEGFA through the PIK3C2α/Akt/HIF-1α pathway in hepatocellular carcinoma. PLoS One 8: e77957. 24194905 10.1371/journal.pone.0077957 PMC3806796

[10] An X, Xu G, Yang L, Wang Y, Li Y, et al (2014) Expression of hypoxia-inducible factor-1α, vascular endothelial growth factor and prolyl hydroxylase domain protein 2 in cutaneous squamous cell carcinoma and precursor lesions and their relationship with histological stages and clinical features. J Dermatol 41: 76–83. 24354513 10.1111/1346-8138.12314

[11] Shi D, Xie F, Zhang Y, Tian Y, Chen W, et al (2014) TFAP2A Regulates Nasopharyngeal Carcinoma Growth and Survival by Targeting HIF-1α Signaling Pathway. Cancer Prev Res (Phila) 7: 266–277. 24335623 10.1158/1940-6207.CAPR-13-0271

[12] Soleas GJ, Diamandis EP, Goldberg DM (1997) Wine as a biological fluid: history, production, and role in disease prevention. J Clin Lab Anal 11: 287–313. 9292395 10.1002/(SICI)1098-2825(1997)11:5<287::AID-JCLA6>3.0.CO;2-4 PMC6760744

[13] Singh CK, George J, Ahmad N (2013) Resveratrol-based combinatorial strategies for cancer management. Ann N Y Acad Sci 1290: 113–121. 23855473 10.1111/nyas.12160 PMC3713511

[14] Aziz MH, Kumar R, Ahmad N (2003) Cancer chemoprevention by resveratrol: in vitro and in vivo studies and the underlying mechanisms. Int J Oncol 23: 17–28. 12792772

[15] Wen D, Huang X, Zhang M, Zhang L, Chen J, et al (2013) Resveratrol attenuates diabetic nephropathy via modulating angiogenesis. PLoS One 8: e82336. 24312656 10.1371/journal.pone.0082336 PMC3849393

[16] Shin T, Kuboki S, Huber N, Eismann T, Galloway E, et al (2008) Activation of peroxisome proliferator-activated receptor-gamma during hepatic ischemia is age-dependent. J Surg Res 147: 200–205. 18498870 10.1016/j.jss.2008.02.004 PMC2737330

[17] Ito K, Ozasa H, Noda Y, Koike Y, Arii S, et al (2008) Effect of non-essential amino acid glycine administration on the liver regeneration of partially hepatectomized rats with hepatic ischemia/reperfusion injury. Clin Nutr 27: 773–780. 18692283 10.1016/j.clnu.2008.06.012

[18] Papadopoulos D, Siempis T, Theodorakou E, Tsoulfas G (2013) Hepatic ischemia and reperfusion injury and trauma: current concepts. Arch Trauma Res 2: 63–70. 24396796 10.5812/atr.12501 PMC3876547

[19] Wang GL, Jiang BH, Rue EA, Semenza GL (1995) Hypoxia-inducible factor 1 is a basic-helix-loop-helix-PAS heterodimer regulated by cellular O2 tension. Proc Natl Acad Sci U S A 92: 5510–5514. 7539918 10.1073/pnas.92.12.5510 PMC41725

[20] Xu LF, Ni JY, Sun HL, Chen YT, Wu YD (2013) Effects of hypoxia-inducible factor-1α silencing on the proliferation of CBRH-7919 hepatoma cells. World J Gastroenterol 19: 1749–1759. 23555163 10.3748/wjg.v19.i11.1749 PMC3607751

[21] Semenza GL, Jiang BH, Leung SW, Passantino R, Concordet JP, et al (1996) Hypoxia response elements in the aldolase A, enolase 1, and lactate dehydrogenase A gene promoters contain essential binding sites for hypoxia-inducible factor-1. J Biol Chem 271: 32529–32537. 8955077 10.1074/jbc.271.51.32529

[22] Vaupel P (2004) The role of hypoxia-induced factors in tumor progression. Oncologist 9: 10–17. 10.1634/theoncologist.9-90005-10 15591418

[23] Semenza GL (2003) Targeting HIF-1 for cancer therapy. Nat Rev Cancer 3: 721–732. 13130303 10.1038/nrc1187

[24] Michalopoulos GK (2007) Liver regeneration. J Cell Physiol 213: 286–300. 17559071 10.1002/jcp.21172 PMC2701258

[25] Wu SL, Yu L, Jiao XY, Meng KW, Pan CE (2006) The suppressive effect of resveratrol on protein kinase C theta in peripheral blood T lymphocytes in a rat liver transplantation model. Transplant Proc 38: 3052–3054. 17112897 10.1016/j.transproceed.2006.08.150

[26] Gedik E, Girgin S, Ozturk H, Obay BD, Ozturk H, et al (2008) Resveratrol attenuates oxidative stress and histological alterations induced by liver ischemia/reperfusion in rats. World J Gastroenterol 14: 7101–7106. 19084917 10.3748/wjg.14.7101 PMC2776840

[27] Tseng SH, Lin SM, Chen JC, Su YH, Huang HY, et al (2004) Resveratrol suppresses the angiogenesis and tumor growth of gliomas in rats. Clin Cancer Res 10: 2190–2202. 15041740 10.1158/1078-0432.ccr-03-0105

[28] Yu HB, Zhang HF, Zhang X, Li DY, Xue HZ, et al (2010) Resveratrol inhibits VEGF expression of human hepatocellular carcinoma cells through a NF-kappa B-mediated mechanism. Hepatogastroenterology 57: 1241–1246. 21410066

[29] Cao ZX, Fang J, Xia C, Shi XL, Jiang BH (2004) Trans-3,4,5′-trihydroxystibene inhibits hypoxia-inducible factor-1α and vascular endothelial growth factor expression in human ovarian cancer cells. Clin Cancer Res 10: 5253–5263. 15297429 10.1158/1078-0432.CCR-03-0588

[30] Zhang Q, Tang X, Lu QY, Zhang ZF, Brown J, et al (2005) Resveratrol inhibits hypoxia-induced accumulation of hypoxia-inducible factor-1alpha and VEGF expression in human tongue squamous cell carcinoma and hepatoma cells. Mol Cancer Ther 4: 1465–1474. 16227395 10.1158/1535-7163.MCT-05-0198

